# Supplementary material for: Are carbon emissions trading and green financial instruments synergistic? -Comprehensive quantitative research based on content analysis
Source: PLoS One. 2024 Mar 7;19(3):e0298601. doi: 10.1371/journal.pone.0298601 (PMC10919601; doi:10.1371/journal.pone.0298601)
Supplement: S1 Data — (ZIP) [file pone.0298601.s001.zip › Data/地级市Z_F工作报告.zip!/┴╔─■/╜⌡╓▌╩╨/└┤╫╘╣1⁄2╓┌║┼í╛┬φ┐╦╩2╛▌═°í┐_2016.docx]

各位代表：

　　现在，我代表市政府向大会报告工作，请予审议，并请市政协各位委员提出意见。

　　一、“十二五”时期和2015年工作回顾

　　“十二五”时期，在省委、省政府和市委的正确领导下，全市上下奋力拼搏、攻坚克难，锦州经济社会发展取得新的成就。

　　经济实力稳步提升。全市地区生产总值年均增长8.2%，人均地区生产总值超过6700美元;固定资产投资年均增长9.5%；规模以上工业增加值年均增长7.4%；城乡居民人均可支配收入年均分别增长8.9%和10.2%。

　　产业结构逐步优化。三次产业比由2010年的16.6:47.6:35.8调整为15.6:43.4:41.0。农业产业化进程加快，综合生产能力持续提升。工业整体实力增强，光伏、汽车零部件、精细化工等新兴产业不断壮大。现代物流、金融、旅游、会展、电商等现代服务业快速发展，城区服务业集聚区建设成效明显。民营经济对全市经济贡献率达到75%。

　　改革开放步伐加快。大力简政放权，行政审批制度改革走在全省前列。国资国企、商事制度、医药卫生、土地流转等重点领域和关键环节改革不断深化。对外开放广度和深度逐步拓展，连续三年承办辽宁台湾周，成功举办2013中国·锦州世界园林博览会。

　　城乡环境明显改善。锦凌水库建成蓄水。城市供水、供电和燃气用户普及率达到90%以上，集中供热率达到88%，污水处理率达到85%。城市建成区绿化覆盖率达到42%。农村环境治理效果显著。新型城镇化步伐加快，常住人口城镇化率达到54.5%。

　　民生保障得到加强。社会就业稳定增加，社保体系日趋完善，保障性住房建设扎实推进。教育资源不断优化，城乡基层医疗服务体系基本建成，公共文化服务体系不断健全。连续五年获得安全生产先进市。社会和谐稳定局面不断巩固。

　　“十二五”时期，全市经济社会发展取得的成就，令人欣喜和振奋，为辽西区域性中心城市建设奠定了坚实基础。

　　各位代表，刚刚过去的2015年，在市委的正确领导下，我们主动适应经济发展新常态，统筹推进稳增长、调结构、促改革、惠民生、防风险各项工作，保持了全市经济社会的平稳发展。

　　初步预计，全市实现地区生产总值1357.5亿元，固定资产投资782.1亿元，一般公共预算收入75.5亿元，社会消费品零售总额597.7亿元，城镇和农村常住居民人均可支配收入分别达到27040元和12599元。各项主要经济指标增速均高于全省平均水平。

　　（一）滨海新区和松山新区先导区建设步伐加快

　　积极融入“一带一路”发展倡议。锦州港作为中蒙铁路出海口获得国家支持，沈铁宏运集装箱海铁联运等项目投入运营。龙栖湾港区完成投资28亿元，油气储运等项目前期快速推进。锦州湾机场如期通航。

　　滨海新区先导示范作用不断增强。大学城初具规模，渤海大学滨海校区投入使用，辽宁农经校、辽宁理工职业学院本科校区主体竣工，锦州医科大学、锦州师专整体搬迁工作全面启动。中谷琪珑临港物流园、万得汽车电器产业园等项目开工建设，新能源汽车、海尔物流园等项目顺利推进。城中村改造步伐加快。商场、医院等城市服务功能日益完善。

　　松山高新区晋升为国家级高新区。大学科技园起步区开工建设，军民融合产业园规划建设全面启动。中科院沈阳国家技术转移中心锦州中心挂牌，中科院上海高等研究院科技成果转化服务综合信息平台落户高新区。奥鸿生物医药产业园、环宇科技集成电路产业园等一批高新技术产业项目开工建设。

　　（二）工业经济实现稳定增长

　　狠抓运行和项目建设。坚持市领导包扶重点企业，强化百户重点企业运行分析和跟踪服务，举办地工产品销售、银企对接等活动11次，切实帮助企业解决实际困难。表彰选树50户功勋企业，激励企业为稳增长做贡献。全年实施1000万元以上工业项目586个，天桥钼业、睿阳电子等100个项目竣工投产。“6921”工程扎实推进，石化及精细化工等6大产业集群实现销售收入2700亿元以上。

　　积极推进大众创业、万众创新。出台22条鼓励创业、创新政策措施。新认定国家高新技术企业7家、省级产业技术创新战略联盟2家，实施国家引智项目4个，12项科技成果获省科学技术奖。支持民营经济发展，“助保贷”业务惠及96户中小企业，“金融进园区”活动直接为企业融资12.3亿元。“智慧城市”建设全面启动。

　　大力实施品牌战略。创市以上名牌产品8个、著名或驰名商标17个，新认定“专精特新”产品（技术）13种。中信锦州金属、龙栖湾化纤成为国家和省级两化融合管理体系贯标试点企业。

　　(三)县域经济和现代农业取得新进展

　　四县（市）新开工2000万元以上项目189个，其中亿元以上35个、10亿元以上4个。黑山农机装备制造、义县新型冶金材料等县域产业集群累计实现销售收入305亿元。北镇特色文化旅游休闲集聚区获评省级服务业集聚区。

　　积极开展抗旱自救，全市粮食总产量达到45亿斤。黑山蛋鸡养殖量和北镇葡萄鲜储量居全国第一，凌海生猪、义县奶牛“一县一业”格局初步形成。

　　新增农产品加工企业26家、农业产业化龙头企业50家。新增改建设施农业10万亩、国家级粮油高产示范区60个、节水灌溉25.7万亩。兴办各类农民合作社746个。耕种收综合机械化水平达到80%。盘活存量土地413公顷，清理批而未供和闲置土地782公顷。

　　大力开展农村环境建设年活动，建成宜居示范村121个、宜居达标村401个，农村环境面貌有了较大改善。新型城镇化建设扎实推进。

　　(四) 城区经济和现代服务业快速发展

　　扎实推进服务业发展四年行动计划。滨海新区电子商务基地获批国家电子商务示范基地，锦州港物流集聚区晋升省级现代服务业集聚区，辽西小商品·古玩商贸流通集聚区、古塔商贸集聚区获评省优秀公共服务平台。万达广场、银河广场大润发旗舰店等项目开工建设，台湾百脑汇、太阳广场等项目主体竣工，鼎沐大厦、盛世东方汽车销售服务园二期等项目投入使用。夜经济、楼宇经济等业态快速兴起，大连港辽西总部经济大厦开工建设。会展业成交额突破50亿元。出台27条支持政策，举办春夏秋三季房交会，商品房销售额达到77亿元。农产品现代流通体系建设步伐加快，发展“新网工程”配送中心15个。黑山励家中国北方杂粮交易中心年交易额突破10亿元，“北方粮”电商交易平台即将在京启动。

　　全力打造辽西区域金融中心。华夏银行、大通证券在锦设立分支机构，永安财险锦州分公司即将开业，锦银金融租赁有限公司获中国银监会批准。锦州银行在香港联交所上市，辽工维森等4户企业“新三板”挂牌。与省国开行、省农发行签订战略合作协议。全市新增贷款154亿元、非信贷融资493亿元。

　　旅游业蓬勃发展。东方华地城温泉及冰雪项目正式运营，龙海馨港、汤水冬怡等项目开工建设。两列列车冠名“锦州号”。成功举办全国红色旅游高峰论坛和锦州首届冰雪节。东方华地城和九华山晋升国家4A级景区。全市实现旅游收入305亿元，增长12%。

　　（五）各项改革取得新突破

　　成功承办全省简政放权现场会，建设工程项目并联审批经验在全省推广。新一轮简政放权取消、下放行政职权项目59项，取消行政审批前置要件232项、非行政许可审批事项28项。在全国率先开展证明要件清理，取消47项，削减25.4%。新公共行政服务中心建成投入运行，实现审批部门成建制应进必进、一个窗口审批和一站式全流程服务。公布政府部门权责清单。完成清理行政审批中介服务事项工作。

　　推进新一轮机构改革，理顺服务贸易和招商体制机制，组建商务局、经济合作局。北镇沟帮子、义县七里河产业园区体制机制创新试点工作完成。市属党政机关所属企业脱钩改制工作顺利实施。驻锦央企“三供一业”分离移交工作全面启动。工商登记制度改革成效显著，新登记各类市场主体2.6万户。完成“个转企”1707户。林权主体改革基本完成，事业单位分类改革、国有文艺院团等改革稳步推进。

　　(六) 对外开放水平不断提升

　　招商引资和产业园区空间建设“百日会战”取得预期成效。共包装项目420个、洽谈308个、签约159个、开工134个，超额完成预定目标。10个重点产业园区完成基础设施建设投入9亿元，新增面积10平方公里，项目承载能力明显提升。全年共组织境内外经贸洽谈活动36次，新引进总投资亿元以上项目120个。

　　全力抓好国发28号文件、央企民企沪企辽宁行、拟列入国家和省“十三五”规划项目的对接工作。古塔老工业区和黑山县八道壕独立工矿区搬迁改造项目纳入国家支持范围，锦凌水库供水工程、主城区地下综合管廊等项目列入国家专项建设债券支持，辽西产业合作与转型升级示范区、松山新区产城融合示范区项目报国家发改委待批。建立全市项目总库和县（市）区、部门项目子库，96个项目纳入省“十三五”专项规划。

　　加强出口基地建设，新增自营进出口经营权企业63家。与长城工业、长征国际等央企军贸对接活动卓有成效。外贸出口13.5亿美元。口岸通关便利化工作稳步推进，锦州口岸获批国家粮食进口指定口岸，锦州港保税物流中心（B型）项目报省政府和沈阳海关待批。

　　(七)城乡规划建设管理水平不断提高

　　加强城市规划建设管理。编制完成主城区综合管廊、城区公厕、户外广告及招牌、松山金融集聚区、渤海大道两侧重点片区等专项规划。健全城市监督管理体制，城区网格化管理全面实行。开展城市规划和城区户外广告招牌、出租车市场专项整治，依法规范管理市区内残疾人代步车，市容市貌明显改观。

　　基础设施建设不断加强。中央南街立交桥、士英街桥洞扩孔改造工程竣工通车。滨河路三期新建工程进展顺利。广州街小凌河大桥、云飞街桥洞扩孔改造工程前期工作加快推进。新建大修城区街路20条，完成小街小巷道路改造56条。768公园、士英公园竣工对外开放。完成主城区7座桥梁亮化工程。大中修县以上公路295公里，建设农村公路候车亭25对。华润锦州电厂2台66万千瓦机组开工建设，锦凌水库附属工程和25万吨净水厂主体工程竣工，政府与社会资本合作（PPP）项目城市综合管廊试验段破土动工。

　　城乡生态环境持续改善。“两河”治理工程城区段如期竣工，一条崭新的生态带、文化带、旅游带、休闲带、安全带惠及广大市民。锦凌水库水土保持和环境保护工程完成，实施移民后期扶持项目35个。凌海大凌河滨河路一期、义县大凌河生态文明示范区一期主体工程竣工。建成绿化示范村94个，人工造林20.6万亩，封山育林22.5万亩，治理水土流失32万亩。大笔架山海岛整治修复工程竣工。完成环保设施提标改造项目45个，整改60家环境违法企业，黄标车及老旧车辆淘汰、燃煤小锅炉拆除等工作有序推进。

　　(八)民生工程和社会事业协调发展

　　扎实开展基础教育建设年活动。北镇市、古塔区义务教育均衡发展通过国家认定，凌海市、太和区通过省审核性评估。新、改建城乡公办幼儿园35所,“三通两平台”建设和90个农村义务教育薄弱学校改造项目基本完成。整合城区职教资源，成立市现代服务学校，完成职业教育布局结构调整。招聘特岗教师167人。表彰首批锦州名师30名。

　　卫计系统专项整治成效明显。医疗市场秩序进一步规范，医疗保障水平不断提高。区域影像中心和检验中心建设加快推进。市妇婴医院综合门诊楼投入使用，市中心医院门诊住院综合楼开工建设。8家县级公立医院完成综合改革。发放居民健康卡157万张。新农合补助标准达到年人均380元。与铁岭、盘锦两市成功实现城镇职工就医异地结算。

　　实施文化惠民工程。新编话剧《张鸣岐》受到广泛好评，以最美村官王桂兰为题材的电影《为了这片土地》荣获4项大奖。成功举办市首届群众文化节。广济寺维修工程获第二届全国“十佳”文物保护工程。建成社区书屋101个。万人文化广场和市京剧团剧场维修改造工程竣工，市文化艺术中心开工建设。新建农民健身工程432套，群众性体育活动深入开展。

　　就业社保等工作卓有成效。实现城镇实名制就业3.98万人，登记失业率控制在4.28%以内。城乡低保和农村五保供养标准分别提高10.7%和15.5%。建成保障性住房3616套。黑山八道壕沉陷区治理工程完成，2031户居民顺利回迁。完成老旧小区综合整治237万平方米，“暖房子”工程30万平方米。城市和农村社区用房平均面积分别达到338平方米、251平方米。市养老综合服务中心主体工程完工。完成新的人力资源市场建设。开通市区至滨海新区公交线路2条。实现国标贫困人口脱贫3.6万人。完成1020户残疾人家庭无障碍设施改造。解决11.4万农村人口饮水安全问题。

　　平安锦州建设迈出新步伐。全面加强立体化社会治安防控体系建设，严厉打击各类违法犯罪活动，突出治安问题得到有效整治。圆满完成“六五”普法任务。第八轮争创全国双拥模范城工作顺利推进。食品安全放心县（市）创建工作全面启动。严格落实主体责任，加大油气管道等隐患排查整治力度，安全生产持续稳定。整合诉求渠道，建立市群众诉求系统服务平台。逐案落实主体责任，信访积案办结率明显提升，信访工作全面加强。

　　(九)政府自身建设进一步加强

　　自觉接受人大法律监督、工作监督和政协民主监督，坚持重大事项向人大报告、政协通报制度。认真办理人大议案、代表建议和政协建议案、委员提案，办复率达100%。

　　扎实开展法治锦州建设年活动，依法规范行政权力运行，全民法治观念不断增强。完善决策机制，科学化民主化法治化决策水平不断提高。加强行政执法监督，行政执法行为日益规范。

　　深入落实党风廉政建设责任制，自觉履行“一岗双责”。推进重点领域政务公开。严格财政预算管理，“三公”经费下降11%，行政成本明显降低。强化对稳增长政策落实及政府重大投资的跟踪审计。出台政府性债务管理办法和风险应急处置预案，公共资源交易、土地收储、规划审批、投融资工作进一步规范。

　　扎实推进“三严三实”专题教育，认真查找不严不实问题，坚持做到边查边改、立行立改。自觉践行省委“八个带头、八个自觉”要求，带头做“讲诚信、懂规矩、守纪律”的模范。进一步强化绩效考评和监督执纪问责，不作为、慢作为、乱作为问题得到有效遏制，政府系统风清气正、干事创业氛围日益浓厚。

　　国防动员、边海防建设、人防和民兵预备役工作不断加强，气象、统计、外事侨务、民族宗教、政务接待、地震监测、住房公积金等工作取得新进展。

　　各位代表！回顾“十二五”和去年的工作，我们深切的感受到，锦州发展建设取得的巨大成就，是省委、省政府和市委正确领导的结果，是全市广大干部群众共同努力的结果。在此，我代表市政府，向全市人民表示崇高的敬意！向人大代表、政协委员，向各民主党派、工商联、无党派人士和各人民团体，向中省直单位，向驻锦部队、武警官兵，向离退休老同志，向所有关心支持锦州发展建设的海内外朋友，表示衷心的感谢！

　　在总结回顾工作的时候，我们也清醒地看到，当前还面临一些困难和问题：一是经济下行压力持续不断，一些指标增速回落幅度较大，部分行业和企业生产经营还存在诸多困难，去产能、去库存任务艰巨；二是固定资产投资后续增长乏力，具有牵动作用的大项目少，转方式调结构任务繁重；三是创新要素聚焦不足，自主创新能力不强，大众创业、万众创新氛围还不够浓厚；四是财政收支矛盾加剧，保障改善民生的压力依然较大；五是一些历史遗留问题亟待解决，社会稳定形势不容乐观；六是党风廉政建设主体责任和监督责任落实得还不够到位，个别部门、个别干部不敢担当、不愿作为，甚至违法乱纪的问题依然存在，政府自身建设还需要进一步加强。对此，我们一定高度重视，着力加以解决。

　　二、“十三五”时期目标任务和2016年主要工作安排

　　各位代表，“十三五”时期是全面建成小康社会的决胜期，是锦州建设辽西区域性中心城市的关键期。我们要看到困难，更要看到希望。经济下行压力持续加大，体制机制弊端制约掣肘发展，稳增长任务更加艰巨，这是不争的事实。但“一带一路”、京津冀协同发展、新一轮东北振兴等国家战略同步实施，则为我们产业转型升级、保持经济稳步增长注入了新的动力。我们坚信，“十三五”时期我们大有可为，我们有基础、有条件、有能力把锦州发展得更好、建设得更美。

　　“十三五”时期发展的主要目标是：经济保持中高速增长，增速不低于全省平均水平。全市地区生产总值年均增长7%左右，一般公共预算收入年均增长5%左右，固定资产投资年均增长7%左右。三次产业比调整为12:44:44。全部完成7.5万贫困人口脱贫任务。结构调整取得重大进展，工业化和信息化深度融合，自主创新能力全面提升，各项改革扎实推进，开放型经济水平大幅提高。文化和社会建设明显加强，生态环境不断改善，城乡居民人均收入与经济发展同步增长，人民生活水平显著提高。打赢爬坡过坎攻坚战，全面建成小康社会，初步形成区域性中心城市发展框架，集聚辐射服务功能充分显现。

　　实现“十三五”奋斗目标，我们必须全面贯彻党的十八大和十八届三中、四中、五中全会精神，按照中央“四个全面”战略布局、习近平总书记“四个着力”和省委、省政府激活“四个驱动”、培育“六个新增长点”的要求，牢固树立创新、协调、绿色、开放、共享的发展理念，深入实施对外开放、创新驱动、以港兴市、工业强市、生态立市战略，注重去产能、去库存、去杠杆、降成本、补短板，加大供给侧结构性改革攻坚力度，全面推进稳增长、调结构、惠民生、防风险各项工作，坚定不移地加快推进辽西区域性中心城市建设。

　　各位代表，今年是实施“十三五”规划的开局之年，也是推进结构性改革的攻坚之年。全市经济社会发展主要预期指标是：地区生产总值增长6%左右，固定资产投资增长6%左右，规模以上工业增加值增长4.5%左右，一般公共预算收入增长4.5%左右，社会消费品零售总额增长8.5%左右，居民消费价格涨幅3%左右，城镇和农村常住居民人均可支配收入均增长7%左右。

　　实现上述目标，我们要深入贯彻中央、省和全市经济工作会议精神，正确把握发展的阶段性特征，积极适应引领经济发展新常态，把稳增长作为重中之重，全面提高发展的质量和效益，确保全市经济社会的平稳健康发展。全年要突出抓好以下九个方面重点工作:

　　（一）以招商引资项目建设年为牵动，加快培育增长新动力

　　紧紧围绕稳增长调结构，进一步加大招商引资力度，大力开展招商引资项目建设年活动。依托产业资源优势，面向日韩、欧美、港澳台、京津冀、长三角、珠三角等重点国家和地区，强力推进以商招商、专业招商和集群招商。继续加强重点产业园区空间建设，完成投资15亿元，新增园区基础设施面积15平方公里，进一步提升项目承载能力。力促大有经济区、汤河子产业区晋升省级经济开发区。实施市级领导包扶重大项目制度，落实县（市）区招商引资主体责任，明确部门招商引资任务职责，建立健全跟踪调度和督查考核机制，确保年内实现域外资金到位额360亿元，引进1000万元以上项目245个，其中亿元以上130个。

　　探索外资并购、企业境外上市等利用外资方式，鼓励国外跨国公司在锦设立分支机构。加快企业“走出去”步伐，积极参与境外资源开发和工程承包。全面落实外贸政策措施，着力解决石化、铁合金等重点行业出口难题。推进同国际知名电商合作，积极培育跨境电子商务平台。加强省级外贸示范基地建设，重点培育锦港物流等一批外贸综合服务企业。积极推进通关便利化，力争锦州港口岸扩大开放获国家批准，保税物流中心（B型）获海关总署批准。

　　（二）以两个先导区率先发展为突破，加快形成产业技术综合竞争优势

　　抢抓“一带一路”倡议机遇。深化同锡盟和蒙古国交流合作，支持蒙古国在锦州港设立海事办事处，力争实现锦州港至二连浩特过境货物海铁联运，加快推进中蒙铁路出海口建设。进一步完善“一港双区”发展规划和功能定位，统筹推进疏港铁路公路建设。投入港建资金15亿元，完成锦州港煤炭码头一期、西部海域防波堤和龙栖湾港区3万吨级航道、起步区码头等工程建设。锦州湾机场力争开通2条新航线。

　　加快建设国家级开发区。利用港口和腹地资源优势，加快推进邈矣石化、龙栖湾港区油气储运等项目前期工作；确保新能源汽车、锦州港油页岩综合利用等项目开工建设，汉拿电机、中铁上海钢构等项目投产达效。加快大学城、科研实验基地和万人客服呼叫中心建设，推进锦州师专整体搬迁。启动通用航空产业园、空港物流园和滨海新区客运站建设。

　　加快建设国家级高新区。完成大学科技园起步区建设，实现10家院校科研机构及企业研发总部入驻，推进北航大学科技园分园等项目落户。中科院上海高等研究院科技成果转化服务综合信息平台投入运营。重点推进高新技术企业孵化基地、国家科技成果转化服务示范基地等项目建设，促进一批重大科技成果就地转化。全面启动军民融合产业园建设。加快完善南站地区工业及生活配套设施。

　　（三）以创新发展带动新型工业化进程，推动工业稳增长、降成本、增效益

　　狠抓工业经济运行。坚持对100户重点企业进行全程运行监测调度，继续实施市领导帮扶企业制度，举办地工产品产销衔接会，有效搭建银企对接平台，着力帮助企业特别是停产半停产企业解决实际困难。

　　加快推进工业结构调整和转型升级。深化工业强市战略，全面实施“6921”、《中国制造2025》提升、传统产业转型升级和质量品牌建设等四大工程。围绕“6921”工程抓龙头、上项目、促配套，力促天龙新材料差别化纤维二期等100个亿元以上项目开工建设，辽宁天合60万吨氟素润滑油等100个项目竣工投产，奥鸿生物医药产业园等100个新兴产业项目加快建设。积极稳妥处置“僵尸”企业，切实抓好去产能工作。全面实施《中国制造2025》锦州工作方案，大力推进智能制造，加强产品和产品生产环节同信息技术的综合集成。切实做好中信锦州金属、龙栖湾化纤“两化融合”试点企业后续贯标工作。改造升级输变电、矿山机械、包装机械等传统产业，不断增强装备制造核心竞争力。以技术中心和龙头企业建设为依托，推进质量品牌建设，创市以上名牌产品10个。加快中小企业公共服务平台建设，完善平台功能和企业数据库，推进民营经济发展。

　　积极打造创新发展新优势。以光伏及电子信息、汽车及零部件、精细化工、生物制药等新兴产业为重点，鼓励支持企业开发“专精特新”产品和技术，促进“互联网+工业”快速发展。积极争取利用省产业（创业）投资引导基金，支持设立以企业为主导的各类产业及创业投资基金。力争每个县（市）区建立1个中小微企业创业基地。支持企业、高校、科研院所直接引进创新人才和创新创业团队。充分利用云计算、大数据等新技术，加快推进“智慧城市”建设。

　　（四）以现代农业和特色产业发展为支撑，做大做强县域经济

　　在全面加快发展农产品深加工产业的同时，各县（市）要结合本地实际，进一步明确产业发展定位。黑山县要在做强特色农业的基础上，重点做好大宗农产品交易中心、农机装备制造、新能源和绿色建材等基地建设。北镇市要努力打造闻名全国、辐射华北的绿色有机农副产品生产基地和交易集散基地，大力培育休闲旅游产业，加快推进石墨科技产业园建设。凌海市要重点发展海洋、旅游、临港临空等产业，及以高新技术为主的装备制造、新材料、生物科技和循环经济。义县要重点发展精细化工、智能制造等产业，做强做大七里河工业园区和国大辉山乳业、春光包装机械等企业。围绕产业定位，加快推进县域项目建设。每个县（市）新上亿元以上项目10个，其中10亿元以上2个。

　　加快现代农业发展。粮食总产量稳定在50亿斤以上。绿色、有机食品生产面积达到60万亩，高效节水灌溉9.7万亩。新增各类农民合作社200家以上。建设生态养殖小区10个，规模化养殖达到65％以上。推进义县粮改饲试点。大力实施“互联网+惠农工程”，加快推进12316云服务、农超对接等公共服务平台，以及农产品安全溯源体系建设。

　　推进新型城镇化建设。全面深化户籍制度改革，有序推进农业转移人口市民化。加大城镇基础设施建设力度，逐步实现基本公共服务常住人口全覆盖。

　　（五）以现代服务业发展为主攻方向，全面激发城区经济发展活力

　　深入实施服务业发展四年行动计划。继续抓好十里商街、南郡天下创意产业园等服务业集聚区建设。大力发展现代物流业，支持综合物流园和龙头企业发展，推进配送中心和公共信息服务平台建设，加快培育综合性现代农产品交易中心和大型专业批发市场，筹建锦州港冷链物流基地。依托滨海电子商务产业基地等企业，打造电子商务公共服务平台，推进“众创空间”建设，带动相关配套产品融合发展，推动传统服务业转型升级。继续办好古玩文化节、北方农展会、国际汽车展等品牌展会。加大商品房去库存力度，促进房地产业平稳健康发展。大力发展信息、教育文体、家政服务、健康养老等新兴服务业，培育新的消费热点。

　　加快旅游强市建设步伐。推进国有旅游企业改组改制，加强区域合作，创新旅游营销模式，大力发展温泉、山川、乡村和生态旅游，办好冰雪节、梨花节、苹果节等节展活动。

　　加快辽西区域金融中心建设。抓好松山金融集聚区和省级科技金融示范区建设。建成全市金融网络服务平台。争取2家域外银行和2家保险证券公司在锦设立分支机构，新增担保公司2家。完成农商行改制。锦银金融租赁有限公司开业运营。启动锦州钛业上市前期工作，争取富山密封等6家企业“新三板”挂牌。

　　加快推进城区经济发展。各城区在大力发展商贸流通、楼宇经济、电子商务、休闲旅游等产业的同时，要立足各自比较优势，突出特色发展、差异化发展。古塔区要重点发展石油化工、电子信息、科教医疗等产业，依托锦凌水库生态资源，大力发展健康养老、观光农业。凌河区要重点发展金融、教育、文化、现代物流和总部经济等重点产业，加快打造城市中央商务区。太和区要重点围绕钛及特种金属、机械制造、包装印刷等产业推进项目建设，加快生产企业主辅分离，积极发展生产性服务业。

　　（六）以推进结构性改革为重点，着力解决制约发展的深层次问题

　　大力简政放权。聚焦束缚大众创业万众创新、企业投资经营和便民服务的突出问题，进一步转变政府职能。做好国家和省取消下放行政审批事项承接落实工作，再取消和下放一批行政审批事项。完善权责清单制度建设，适时发布负面清单。全面推行重大项目“一表制”审批。推进网上审批监管平台建设。进一步取消各类无谓证明和繁琐手续。深化工商登记制度改革，探索试行简易注销、简化名称和经营范围等登记改革。继续抓好个转企工作。整合建设工程交易、招投标、地产交易、产权交易四个中心，彻底实现管办分离。

　　深化国有企业改革。整合重组锦州粮食产业集团。全面做好驻锦央企“三供一业”分离工作。加快现代企业制度建设，全民所有制企业公司化改造达到80%以上。按照省政府统一部署，深化事业单位改革，推进经营性事业单位转企，组建大型企业集团，实行国有资产集中统一经营。

　　深化农业农村改革。引导规范农村土地经营权有序流转，促进适度规模化经营。深化供销合作社综合改革，进一步完善农副产品流通体系。稳妥推进国有林场改革。推广七里河和沟帮子两个产业园区改革经验，全面完成重点产业园区体制机制创新。

　　推动财税体制改革。完善政府预算体系，优化转移支付结构。进一步健全政府性债务风险管理制度，全面加强政府债务化解和风险防范。建立养老保险风险基金，强化基金管理和运营。加快推进公车改革。

　　（七）以建设生态宜居锦州为目标，加大城乡环境规划建设管理力度

　　加快重大基础设施建设。开展城市轨道交通规划和前期工作，编制锦娘路两侧、薛东薛西地块详细规划和城区停车场专项规划。充分利用PPP合作模式，开工建设广州街跨小凌河大桥、城市综合管廊一期等重点工程。建设小凌河人行步道桥，新建、大修改造城区道路51条。实施小凌河沿岸及城市主要干道两侧楼体亮化工程。维修改造市客运总站，建设农村公路候车亭62个，续建渤海物流园区二期工程。

　　继续实施碧水、青山、蓝天、沃土工程。开工建设南山公园，改造北湖公园。推广城区立体绿化，推进园林绿化养护市场化试点。巩固“两河”治理成果，抓好后期运营管护。加快城乡垃圾处理设施建设，开工建设混合垃圾填埋场，中科生活垃圾及污泥焚烧发电项目建成投入使用，加快南山陈化垃圾处理，促进垃圾资源化利用。人工造林16万亩，封山育林12万亩，治理水土流失24万亩。建立医巫闾山和北普陀山森林防火预警系统。加强大气污染防治，完成节能降耗和淘汰落后产能任务。加快华润锦州电厂2台66万千瓦机组建设，拆除燃煤小锅炉383台。推进淘汰黄标车和老旧车辆。集约节约利用土地。实施土壤污染治理与修复工程。

　　开展海岸带综合治理。集中整治非法侵占岸线资源、非法用海、非法捕捞、污染海洋环境等违法行为。集约节约利用海域资源，积极申报国家级海洋生态示范区。加快发展现代渔业，推进海洋牧场建设。新建大有海防堤9.8公里。

　　巩固提升城乡环境建设整治成果。加快城市管理指挥中心建设，全面推行网格化管理，提高城市精细化管理水平。进一步完善基础设施，建立保洁长效机制，全面提升村庄亮化、美化和服务管理水平。

　　(八) 以持续增强群众的获得感为出发点和落脚点，切实抓好民生保障和社会事业

　　让锦州人民的日子越过越好，是政府的职责所在，也是我们的目标追求，我们要继续带着感情办好各类民生实事。

　　推进创业带动就业。实施稳企援岗政策和高校毕业生创业引领、就业见习计划。启动建设新的市级创业孵化基地。新增就业1.4万人，登记失业率控制在4.5%以内。

　　完善社会保障体系。依法加强社会保险扩面和基金征缴，确保社会保险金按时足额发放。市养老综合服务中心投入使用。市殡仪馆迁建工程开工建设。完成社会救助综合信息平台建设。全面落实困难残疾人“两项补贴”政策。对850户贫困残疾人家庭进行无障碍设施改造。大力实施精准扶贫，实现农村脱贫2万人。

　　持续推进基础教育建设年工作。促进学前教育公益普惠发展。深入开展义务教育均衡发展县（市）区创建工作。有效实施中小学校长、教师轮岗交流。促进普通高中优质、特色、多样化发展。巩固中等职业学校整合成果，加快现代职业教育发展。推进锦州卫校与锦州师专合并工作。

　　扎实开展文化建设年活动。加快构建公共文化服务体系。大力发展文化产业。推进市文化艺术中心建设。实施专业艺术人才、舞台精品、高雅艺术惠民工程。开展周末文化广场、“凌河之夏”演出季、文化志愿者“五进”等活动，组织大型公益演出100场。实施辽沈战役纪念馆基本陈列升级改造。

　　推进健康锦州建设。加快市中心医院门诊住院综合楼建设，医学影像中心、检验中心、生殖中心建成投入运营。启动市紧急医疗救援中心迁建工作。普及应用居民健康卡。整合乡镇卫生计生资源，推进城乡分级诊疗。新农合政府补助标准达到400元以上、参合率稳定在90%以上。全面实施一对夫妻生育两个孩子政策。

　　大力发展体育事业。承办省第七届老年人运动会，举办市第二届全民运动会。新建市足球运动学校。完成滨海体育中心体育场主体工程、体育工艺及装饰工程建设。推进城区15分钟和农村30分钟健身圈建设。设立体育产业发展引导基金，推进体育产业快速发展。

　　实施畅通工程。编制市区交通拥堵疏解与管理实施方案。推进海绵型城市建设，实施雨污分流改造，新增海绵型透水林荫停车场8000平方米。改造北京路转盘、福德转盘等5处交通拥堵路口，加快建设公交专用道等配套设施。购置新能源公交车110辆。维修改造县级公路78公里，大中修普通公路98公里，新建改造农村公路669公里。

　　改善群众基本生活条件。规划建设新的热源工程，改造老旧供热管网20公里。完成棚户区改造3728套，农村危房改造2800套。全面完成锦凌水库移民安置。整治老旧小区330万平方米，完成“暖房子”工程30万平方米。解决农村8.1万人饮水安全问题。

　　开展食品药品安全专项整治。加大监管力度，启动规范化建设，严厉打击食品药品制假售劣行为，落实“黑名单”制度。加强食品经营小作坊规范化监管。开展食品安全放心县（市）建设。

　　深入推进平安锦州建设。创新社会管理，提升立体化社会治安防控水平，严厉打击各类违法犯罪。扎实开展“七五”普法，扩大法律援助覆盖面。切实加强民族宗教工作。做好民兵预备役和人防工作，全力为部队办实事解难题，巩固第八轮双拥模范城创建成果。强化政府应急、网络安全和气象防灾减灾体系建设。推进群众诉求系统服务平台稳定运行。依法处理各类信访，努力解决信访积案和历史遗留问题。严格落实主体责任和监督责任，深入开展安全生产隐患排查整治，坚决遏制重特大安全事故发生，确保社会和谐稳定。

　　（九）以提升行政效能和服务水平为核心，进一步强化政府自身建设

　　新常态新任务，给政府工作提出了新标准新要求，我们将进一步加强法治政府、廉洁政府和服务型政府建设，大力提升政府行政效能、公信力和服务水平。

　　全面推进依法行政。自觉接受市人大及其常委会的法律监督、工作监督，认真落实市人大及其常委会各项决议决定，坚持向人大及其常委会报告工作。主动加强与市政协的联系，积极支持市政协履行政治协商、民主监督和参政议政职能。全力办好人大代表建议和政协委员提案，广泛听取各民主党派、工商联、无党派人士和人民团体的意见建议，不断提高政府工作法治化、规范化水平。

　　始终坚持勤政为民。切实把群众的安危冷暖放在心上，真诚倾听群众呼声，真实反映群众愿望，真情关心群众疾苦，真心解决群众困难。深化政务公开，畅通政务信息，加强软环境建设，全面提升服务水平。坚持说实话、办实事、求实效，确保做到言必信、行必果，以实实在在的工作取信于民。

　　切实加强党风廉政建设。巩固“三严三实”专题教育成果，积极落实党风廉政建设主体责任和监督责任，严格遵守《准则》和《条例》，坚持“讲诚信、懂规矩、守纪律”，努力做到忠诚、干净、担当。全面加强工作督查、行政监察和审计监督，自觉把纪律规矩挺在前沿，坚决遏制违法乱纪行为的发生。

　　大力弘扬真抓实干作风。进一步完善政府绩效考评机制，加强调度、通报和奖惩，确保政令畅通、令行禁止。始终满怀发展激情，以时限倒逼进度，以目标倒逼责任，努力把职责范围内事情办实办好，确保政府各项工作经得起实践、人民和历史的检验。

　　各位代表，我们已经踏上“十三五”新的征程。神圣使命激励我们勇往直前，人民期望不容我们丝毫懈怠。让我们在市委的坚强领导下，团结带领全市人民，以更加昂扬的斗志，更加坚定的步伐，奋力拼博、开拓进取，勇于担当、主动作为，为全面建成小康社会，加快辽西区域性中心城市建设而努力奋斗！
